# Modeling Powassan virus infection in *Peromyscus leucopus*, a natural host

**DOI:** 10.1371/journal.pntd.0005346

**Published:** 2017-01-31

**Authors:** Luwanika Mlera, Kimberly Meade-White, Greg Saturday, Dana Scott, Marshall E. Bloom

**Affiliations:** 1 Laboratory of Virology, National Institute of Allergy and Infectious Diseases, National Institutes of Health, Rocky Mountain Laboratories, Hamilton, Montana, United States of America; 2 Rocky Mountain Veterinary Branch, National Institute of Allergy and Infectious Diseases, National Institutes of Health, Rocky Mountain Laboratories, Hamilton, Montana, United States of America; Colorado State University, UNITED STATES

## Abstract

The tick-borne flavivirus, Powassan virus (POWV) causes life-threatening encephalitis in humans in North America and Europe. POWV is transmitted by ixodid tick vectors that feed on small to medium-sized mammals, such as *Peromyscus leucopus* mice, which may serve as either reservoir, bridge or amplification hosts. Intraperitoneal and intracranial inoculation of 4-week old *Peromyscus leucopus* mice with 10^3^ PFU of POWV did not result in overt clinical signs of disease. However, following intracranial inoculation, infected mice seroconverted to POWV and histopathological examinations revealed that the mice uniformly developed mild lymphocytic perivascular cuffing and microgliosis in the brain and spinal cord from 5 to 15 days post infection (dpi), suggesting an early inflammatory response. In contrast, intracranial inoculation of 4-week old C57BL/6 and BALB/c mice was lethal by 5 dpi. Intraperitoneal inoculation was lethal in BALB/c mice, but 40% (2/5) of C57BL/6 mice survived. We concluded that *Peromyscus leucopus* mice infected i.c. with a lethal dose of POWV support a limited infection, restricted to the central nervous system and mount an antibody response to the virus. However, they fail to develop clinical signs of disease and are able to control the infection. These results suggest the involvement of restriction factors, and the mechanism by which *Peromyscus leucopus* mice restrict POWV infection remains under study.

## Author summary

Powassan virus (POWV) is a neuroinvasive tick-borne flavivirus (TBFV), which causes life-threatening encephalitis in humans in North America and Europe. The virus is persistently maintained in a natural cycle involving infected ixodid ticks and small-to-medium sized mammals, such as mice, woodchucks and skunks. Despite conclusive evidence showing that wild rodents play an important role in the maintenance of TBFVs in nature, very little is known regarding the relationship between POWV and its natural mammalian hosts. We selected *Peromyscus leucopus*, one of the most abundant mixed forest rodent in the USA, as a natural host model for studying POWV infection. Challenging this mouse species with POWV via different routes of inoculation did not result in obvious clinical signs of disease, suggesting resistance, which may be associated with virus restriction factors. The model will be useful for studies aimed at understanding how the animals restrict POWV without clinical signs of disease.

## Introduction

Powassan virus (POWV) is a tick-borne flavivirus (TBFV) belonging to the tick-borne encephalitis virus (TBEV) serogroup. POWV is closely related to the deer tick virus (DTV), also known as POWV lineage II, and the two are the only TBFVs in the TBEV serogroup known to circulate in North America. Current information suggests that the incidence of POWV, particularly lineage II, is increasing in the USA [1]. Despite the availability of a highly effective vaccine, the TBEV serogroup of viruses is responsible for up to 15 000 infections annually in Europe, leading to life-threatening encephalitis and death in close to 40% of infected cases depending on the TBEV strain [2–4]. POWV was first described in 1958 in a fatal case of encephalitis in a 5 year old boy in the small town of Powassan, Ontario, Canada [5]. Since then, POWV has continued to cause sporadic infections in the USA, with cases reported in several states, such as New York State, New Hampshire and Massachusetts [1, 6–11]. POWV is also known to cause human disease in eastern regions of Russia [9].

Transmission of TBFVs to humans is mainly through tick bites, but alimentary infection through the consumption of unpasteurized milk and/or milk products obtained from infected domestic animals in endemic regions is also well documented [12–14]. Although POWV has been isolated from *Dermacentor andersoni* ticks, the blacklegged hard-bodied ticks, *Ixodes scapularis* and *Ixodes cookei*, are the principal vectors responsible for transmitting POWV in North America, and *Hemaphysalis longicornis* is known to transmit the virus in East Asia [1, 15–17]. Field studies have shown that the prevalence of TBFVs in ticks from different geographic locations is variable. For example, the prevalence of Powassan virus in *Ixodes scapularis* in the same area in the state of Connecticut rose from 0% in 2008 to 3.9% in 2010 [18]. A study conducted in Switzerland showed that 0.16–11.11% of *Ixodes ricinus* ticks were positive for TBEV by RT-PCR [19].

Once infected, ticks harbor the virus at each developmental stage for the duration of their lives, which may span several years, and they can also transmit the TBFV transovarially [20–22]. Through a process known as “co-feeding”, infected ticks feeding in close proximity on the same host can rapidly transmit TBFVs to each other via migratory cells in the skin [21, 23–25]. Thus, the ticks play a critical role in the maintenance of TBFVs in nature by direct horizontal tick-to-tick transmission.

The wild animal species responsible for much of TBFV transmission are the same species from which the vectors naturally obtain blood meals, i.e., small to medium-sized mammals, such as white-footed mice (*Peromyscus leucopus*), striped field mice (*Apodemus agrarius*), skunks (*Mephitis mephitis*) and woodchucks (*Marmota momax*) [1, 26–30]. The detection of viral RNA or isolation of TBFVs from wild rodents is substantive evidence implicating these mammals as reservoirs or amplification hosts. For example, the TBEV strain A104 was isolated from the brain of a wild-caught yellow-necked mouse, *Apodemus flavicolis*, in Austria [31]. In Hokkaido, Japan, the TBEV strains Oshima 08-As and Oshima-A-1 were isolated from spleens of *Apodemus speciosus*, and Oshima-C-1 from the gray-backed vole *Clethrionomys rufocanus* [32, 33]. Researchers in South Korea also reported PCR detection as well as isolation of TBEV from lung and spleen tissue dissected from wild *Apodemus agrarius* mice [30]. A group in Finland detected TBEV RNA in mouse brains, but some mice that had viral RNA were seronegative [34].

Additional and surrogate evidence suggesting exposure to TBFVs in wild rodents includes the detection of anti-POWV antibodies in wild-caught *Peromyscus truei* and *Peromyscus maniculatus* in New Mexico; *Myodes rutilus* in Siberia and Alaska; and *Myodes gapperi* in Southern Alaska [9]. Bank voles, *Myodes glareolus*, wild-caught in a small TBEV focus area in Hungary had higher seropositivity rates of 20.5%, than a combined *Apodemus flavicolis* (3.7%) and *Apodemus agrarius* (4.6%) [35]. The difference could be the behavioral result of less mobility of lactating bank voles, which become easy targets for questing TBEV-infected ticks [35].

In spite of this unequivocal evidence that wild small-medium sized mammals play a crucial role in the biology and ecology of the TBFVs, very little is known about how these animals actually respond when exposed to the viruses. The inbred laboratory mouse strain BALB/c is a well-characterized model for studying Powassan virus infection and has been shown to suffer severe neurological disease [36]. Using this model, it has been established that death may occur via respiratory insufficiency, and that yet-undefined component(s) of tick salivary gland extract affects the course of POWV infection [37, 38].

*Peromyscus leucopus* and the congeneric deer mice (*Peromyscus maniculatus*) are the most abundant mixed-forest rodents in eastern USA [39], where most of POWV infections have been reported. After observing that inoculation of POWV (lineage II) caused no overt disease in adult *Peromyscus leucopus* (*P*. *leucopus*) mice, Telford et al. suggested these mice could be reservoirs of POWV [40]. However, limited conclusions regarding pathogenesis in these mice could be drawn from these studies. A recent review also cites resistance or tolerance to POWV in *Peromyscus* mice, suggesting that the species may act as natural reservoirs [41]. Therefore, we decided to rigorously model POWV infection in this natural host. Intraperitoneal (i.p.) and intracranial (i.c.) inoculation of these mice resulted in no overt clinical signs of disease. However, lesions suggestive of mild inflammation were observed in the brains and spinal cords of i.c.-inoculated mice that were accompanied by limited virus replication in the olfactory lobe of the brain. In contrast to the 2 strains of laboratory mice, the *P*. *leucopus* mice were able to limit the infection and did not suffer clinical disease.

## Methods

### Virus

POWV (a kind gift from Dr. Robert Tesh, World Reference Center for Emerging Viruses and Arboviruses, University of Texas Medical Branch) was triple-plaque purified from a primary stock that had been passaged 6 times in Vero cells (ATCC). The virus was semi-purified by ultracentrifugation in a SW28 rotor (Beckman-Coulter) at 131 000 *x* g over a 20% sucrose cushion at 4°C and resuspended in serum-free Dulbecco’s Modified Eagle Medium (sfDMEM; Life Technologies). Sanger sequencing indicated that the nucleotide sequence of the POWV genome was 99.99% identical to the LB strain. Only 10 nucleotide sequence differences were observed between our POWV strain and that of the LB strain in GenBank (Accession number L06436), leading to 5 amino acid sequence differences in NS1 (L838F), NS3 (N2078T), NS4B (A2444S and A2457V), and NS5 (G2670E).

### Ethics statement

The Rocky Mountain Laboratories (National Institute of Allergy and Infectious Diseases, National Institutes of Health) Animal Care Use Committee reviewed and approved the animal study protocol (ASP), number 2014-012-E. The ASP adhered to the National Institutes of Health Guidelines, the Public Health Service Policy on Humane Care and Use of Laboratory Animals, the United States Department of Agriculture’s Animal Welfare Act, and the Guide for the Care and Use of Laboratory Animals.

### Mice and inoculations

All the mice used in this study were 4 weeks old, and all animal experiments were performed in animal biosafety level-3 (ABSL3) facilities. *P*. *leucopus* mice were bred from a colony at the Rocky Mountain Laboratories’ animal facility. The colony was established from *P*. *leucopus* mice, which were wild-caught in North-Western USA more than 13 years ago. Sentinel monitoring of the colony is performed quarterly and the colony is negative for all tested murine pathogens. BALB/cAnNHSd mice were purchased from Harlan Laboratories and C57BL/6J mice were supplied by Jackson Laboratories. All mice were housed in individually ventilated cage systems with a 12:12 light-dark cycle with water and food provided *ad libitum*.

Intraperitoneal inoculations were performed with POWV doses between 10^2^ and 10^8^ PFU in 100 μl sfDMEM using a 25 gauge x ½" needle. Intracranial infections were done with 10^3^ PFU of POWV in 50 μl sfDMEM. Control mice for the different routes were inoculated with equivalent volumes of serum-free DMEM. Experimental end points were set at 28 days post infection.

### Total RNA extraction and qPCR

The mouse organs that were harvested at necropsy for total RNA extraction were blood, brain, kidney, liver, cervical lymph nodes and spleen. Blood was centrifuged to remove serum and the cell pellet was used for total RNA extraction. Harvested mouse organs were homogenized in 1 mL of TRIzol (Invitrogen) by vigorous beating with steel or ceramic beads. Total RNA extraction was performed using an RNeasy kit (Qiagen) according to the RNeasy manufacturer’s protocol.

A microgram of total RNA extracted from each organ was used to synthesize cDNA using a SuperScript VILO cDNA synthesis kit (Invitrogen, Life Technologies) according to the manufacturer’s instructions. The qPCR reaction was made up of 1X POWV-specific forward (GCACGGACCTCTATGTGTATTC) and reverse (AACTGGTCCTCTCACTGTAGTA) primers, probe (TAGTGCAGTGGAAAGAAGCGCAGA), 1X Platinum qPCR SuperMix-UDG with ROX buffer (Invitrogen), H_2_O and 2 μl cDNA. The qPCR was analyzed on a 7900HT Fast Real Time PCR machine (Applied Biosystems). A plasmid containing an insert with the target POWV sequence was serially diluted and used for the standard curve from which the genome copy numbers in various mouse organs were extrapolated.

### Serological assays

An in-house IgG ELISA was used to detect anti-POWV antibodies in the serum of infected and control mice under BSL3 conditions. The antigen was generated by infecting 2 x 10^6^ Vero cells (ATCC) in a 75 cm^2^ flask (Nunc) with POWV at a multiplicity of infection of 1. At the onset of the cytopathic effect, the monolayer was washed 3 times with cold phosphate-buffered saline (PBS) followed by the addition of 10 ml of PBS containing 10% borate and 1% Triton X-100. Cell lysis was enhanced by 3 freeze-thaw cycles. The lysate was clarified by centrifugation at 3000 rpm for 20 min. 100 μl of a 1:1000 dilution of the lysate was used to coat wells of a 96-well plate (Nunc) overnight at 37°C in a humidified chamber. Unbound antigen was removed followed by washing the wells twice with PBS containing 0.025% Tween 20 (wash buffer). Blocking was performed with a blocking solution of 0.05% skimmed milk in PBS with 0.05% Tween 20 for 2 h at 37°C. The blocking solution was aspirated followed by washing 4 times with the wash buffer. 8, 5-fold serial dilutions of each mouse serum sample were prepared and 100μl of each dilution was added to appropriate wells and incubated at 37°C for 2 h. A positive anti-POWV antibody was hyper-immune serum of POWV-infected C57BL/6 mice, and ascites fluid from uninfected mice was used as negative antibody control. The diluted serum samples were aspirated after the incubation, followed by washing the wells 6 times with the wash buffer and 100 μl of horseradish peroxidase-labeled anti-*P*. *leucopus* IgG antibody (KPL) was added at 1:1000 dilution and incubated at 37°C for 30 min. For the positive control, an anti-mouse IgG antibody (Dako) was used, diluted to 1:1000 and also incubated at 37°C for 30 min. The secondary antibodies were aspirated and the wells were washed 6 times with the wash buffer, and 100 μl of a 3,3',5,5'-tetramethylbenzidine (TMB) substrate (Sigma-Aldrich) was added and incubated at room temperature for 1 h. The reaction was stopped with 2 M H_2_SO_4_ and color reactions were used to qualitatively call positive or negative results by visual examination.

To perform a focus reduction test (FRNT), sera from i.c.-inoculated *P*. *leucopus* mice were serially diluted 5-fold, starting at 1:10. Serum from uninfected *P*. *leucopus* mice was used as negative controls. The diluted serum samples were incubated with 10^3^ PFU of POWV at room temperature for 30 minutes, followed by infecting confluent Vero cells in 12-well plates and incubating at 37°C with rocking for 1 h. The infecting preparations were aspirated and the wells were washed 3 times with PBS. The cells were overlaid with complete DMEM containing 0.8% methylcellulose (Sigma-Aldrich) and incubated for 3 days. POWV foci were developed and counted using an immunofocus assay as described before [42].

### Histology and *in situ* hybridization assay

Necropsies of mock- and POWV-infected mice were performed under ABSL3 conditions. Harvested tissues were fixed in 10% neutral-buffered formalin for 7 days. Tissues were placed in cassettes and processed with a Sakura VIP-5 Tissue Tek on a 12 h automated schedule using a graded series of ethanol, xylene and ParaPlast Extra. Embedded tissues are sectioned at 5 μm and dried overnight at 42°C prior to staining. Hematoxylin and eosin (H&E) staining was performed using standard procedures.

*In situ* hybridization (ISH) was performed on 5 μm tissue sections. Probes hybridizing to the positive-sense genomic (+) and minus-sense complementary (-)RNA strands were designed and synthesized at Advanced Cell Diagnostics (Hayward, California) to target the region spanning nucleotides 246–1687 and 7894–9335, respectively. The probe for the (-) sense strand was specific for the replicating RNA. Detection of POWV RNA was performed using the RNAscope FFPE assay (Advanced Cell Diagnostics) as previously described [43] and in accordance with the manufacturer’s instructions. Briefly, tissue sections were deparaffinized and pretreated with heat and protease before hybridization with target-specific probes for Powassan virus RNA. Ubiquitin C and the bacterial gene, *dapB*, were used as positive and negative controls, respectively. Whole-tissue sections from selected representative cases were stained for Powassan viral RNA, *UBC* and *dapB* by the RNAscope VS FFPE assay (RNAscopeVS) using the Ventana Discovery ULTRA slide auto staining system (Ventana Medical Systems Inc.).

## Results

### Analysis of neurovirulence of Powassan virus

**Fig 1:**
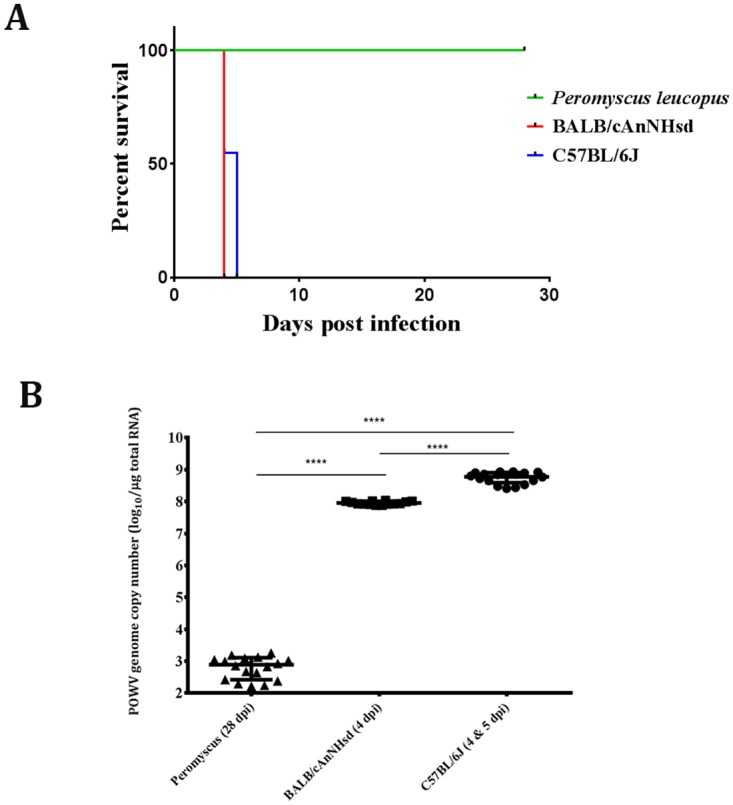
Outcomes of i.c. inoculation of POWV. **(A)** Survival curve of *P*. *leucopus*, BALB/c and C57BL/6 mice after i.c. inoculation. **(B)** Terminal POWV (+)RNA copy numbers in brain homogenates determined by qPCR. 28 dpi copy numbers are presented for *P*. *leucopus* because these mice survived to the experimental end-point, set at 28 days. **** *p*<0.0001 (t-test).

Our first goal was to confirm neurotropism and neurovirulence in 2 laboratory strains of mice, BALB/c and C57BL/6 and then to assess the neurovirulence of POWV in age-matched *P*. *leucopus* mice. Thus, 4-week old BALB/c and C57BL/6 were challenged intracranially (i.c.) with 10^3^ PFU of virus. All 20 BALB/c mice succumbed to disease at 4 dpi, whereas 45% (9/20) of C57BL/6 succumbed at 4 dpi and the rest at 5 dpi (Fig 1A). In both mouse species, the experimental endpoint was preceded by rapid and severe disease, characterized by hunched posture, ruffled fur, hind limb paralysis and severe weight loss. In addition to hind limb paralysis, some C57BL/6 mice also presented fore-limb paralysis.

We determined the level of POWV in the brain of these mice by qPCR analysis, and extremely high copy numbers of POWV RNA genomes were detected in the brains of BALB/c and C57BL/6 mice (Fig 1B). The average genome copy number in the brain homogenates of i.c.-inoculated BALB/c mice was 7 x 10^8^ copies/μg of total RNA, whereas the average genome copy numbers in brain homogenates of i.c.-inoculated C57BL/6 mice was 1 x 10^8^ copies/μg of total RNA (Fig 1B).

Interestingly, qPCR analyses showed that POWV infection of the brain, spread to other organs such as spleens and kidneys after i.c. inoculation. For example, average POWV RNA copy numbers in the C57BL/6 spleens was 3.6 x 10^6^ copies/μg of total RNA (range: 2.3 x104–5.7 x 10^7^ copies/μg of total RNA), and the average in the kidneys was 1.2 x 10^6^ copies/μg of total RNA (range: 6.8 x 10^3^–7.0 x 10^6^ copies/μg of total RNA). The BALB/c spleens contained an average of 7.6 x 10^6^ POWV RNA copies/μg of total RNA (range: 4.4 x 104–9.1 x 10^7^ copies/μg of total RNA) and the kidneys had an average of 1.4 x 10^7^ copies/μg of total RNA (range: 2.0 x 105–9.1 x 10^7^ copies/μg of total RNA). An average of 8.6 x 10^4^ POWV RNA copies/μg of total RNA (range 8.6 x 10^4^–2.5 x 10^5^ copies/μg of total RNA) was detected in the blood of BALB/c mice, whereas an average of 3.3 x 10^6^ copies/μg of total RNA (range: 6.5 x10^4^–2.8 x 10^7^copies/μg of total RNA) was detected in C57BL/6 mouse blood. In summary, i.c. infection of the laboratory strains of mice led to a systemic fatal infection of the nervous system accompanied by widespread permissive viral replication.

We next assessed neurovirulence of POWV for *P*. *leucopus* mice. A total of 20 4-week old *P*. *leucopus* mice were inoculated i.c. with 10^3^ PFU of POWV and observed over a 28 day period. In marked contrast to the BALB/c and C57BL/6 mice, all of the inoculated *P*. *leucopus* mice survived to 28 days (Fig 1A), without showing any clinical signs of disease. These findings indicated that the *P*. *leucopus* mice were resistant to neurological disease induction by i.c. inoculation of a dose of POWV that was lethal for all of the BALB/c and C57BL/6 mice.

Although *P*. *leucopus* mice challenged with POWV via i.c. inoculation did not show any clinical signs of disease, viral RNA was detected by qPCR in their brain homogenates at the experimental end point of 28 dpi (Fig 1B). The average POWV RNA genome copy number in *P*. *leucopus* mouse brains was 778 copies/μg of total RNA. Thus, limited POWV replication took place in the brains of i.c.-inoculated *P*. *leucopus* mice, but this did not lead to obvious clinical disease.

### Kinetics of POWV replication in *P*. *leucopus* mice

**Fig 2:**
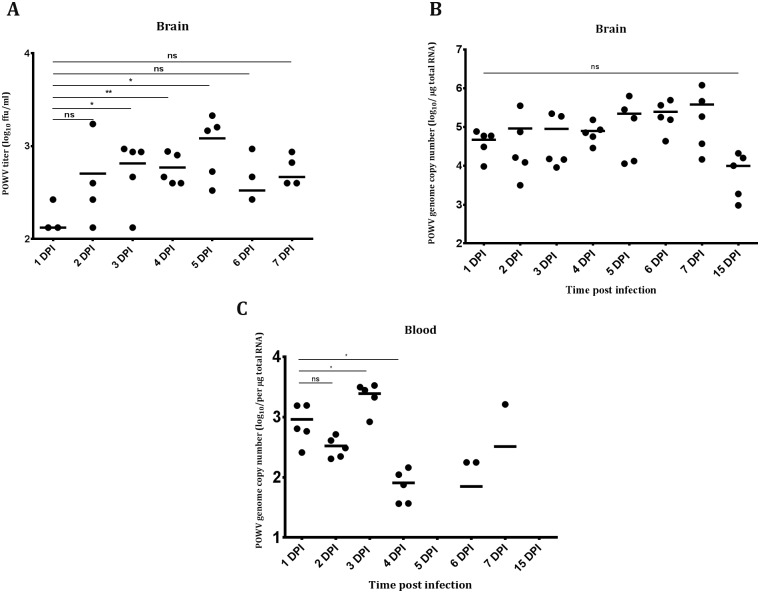
POWV replication kinetics in *P*. *leucopus* following i.c. inoculation. **(A)** POWV titers from brain homogenates harvested from i.c. inoculated *P*. *leucopus* determined by immunofocus assay. **(B)** POWV (+)RNA copy numbers in brain after i.c. inoculation. **(C)** POWV (+)RNA copy numbers in blood after i.c. inoculation. * *p*<0.05; ns: not significant (t-test).

To gain additional insight into the POWV replication kinetics in *P*. *leucopus* mice, we i.c. inoculated 4-week old mice in 8 groups of 5 and euthanized them daily from 1 dpi through to 7 dpi, as well at 15 dpi. We could isolate infectious POWV from the brain homogenates from 1 dpi through to 7 dpi although the virus titer did not increase in a dramatic fashion (Fig 2A). Using qPCR, we determined that the average POWV RNA copy number at 1 dpi was 4.7 x 10^4^ copies/μg of total RNA (Fig 2B). This average steadily peaked to 3.8 x 10^5^ copies/μg of total RNA at 7 dpi, but declined to 1 x 10^4^ copies/μg of total RNA at 15 dpi (Fig 2B). The presence of POWV RNA in the brain was also associated with viral RNA in the blood for the first 4 days post infection (Fig 2C), suggesting short-lived early viremia. These results indicated that POWV was able to initiate a productive, infection in *P*. *leucopus* mice following i.c. challenge, but that the mice were able to limit the infection and remained completely asymptomatic.

### Analysis of neuroinvasiveness of Powassan virus

**Fig 3:**
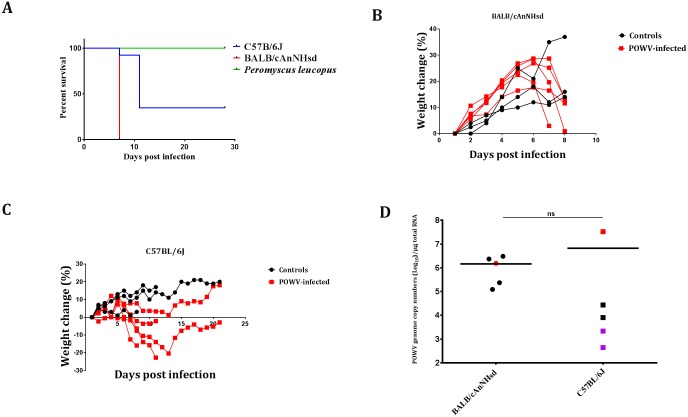
Analysis of i.p. inoculation of POWV in BALB/c and C57BL/6 mice. **(A)** Survival curve for i.p. inoculated BALB/c, C57BL/6 and P. leucopus mice. **(B)** Changes in weights of BALB/c mice after i.p. inoculation of POWV. **(C)** Changes in weights of BALB/c mice after ip inoculation of POWV. **(D)** POWV (+)RNA copy numbers in brains of i.p.-inoculated BALB/c and C57BL/6 mice. The red dot for BALB/c mice denotes RNA copy numbers from the mouse which succumbed first. The red square for C57BL/6 mice denotes the mouse that succumbed at 8 dpi, the black squares represent the mice that succumbed at 11 dpi and the survivors are denoted by the purple squares. ns: not significant (t-test).

Our next aim was to define the neuroinvasiveness of POWV in laboratory mouse strains as well as in age-matched *P*. *leucopus*. Therefore, we inoculated 5 4-week old BALB/c mice intraperitoneally with 10^3^ PFU of virus. BALB/c mice have been previously reported to succumb to POWV encephalitis within 9 days [36], and the mice in our experiments started to show clinical signs of disease i.e., ruffled fur and weight loss at 6 dpi. All of the BALB/c mice progressed to show signs of severe neurological disease, characterized by hind limb paralysis and >15% weight loss and were euthanized by 8 dpi (Fig 3A & 3B). These results confirmed the previous reports that POWV infection in BALB/c mice is neuroinvasive.

We also challenged 5 4-week old C57BL/6 mice i.p. with the same dose of POWV. One mouse was euthanized at 8 dpi due to severe hind limb paralysis, ruffled fur, emaciation and >15% weight loss (Fig 3A & 3C). The other 4 POWV-infected C57BL/6 mice also showed signs of disease at 8 dpi, but the weight loss was variable <15% (Fig 3C). An additional 2 mice developed severe progressive disease and were euthanized at 11 dpi. The 2 surviving C57BL/6 mice recovered and lived without obvious signs of disease until they were euthanized at the experimental end point of 28 dpi (Fig 3A).

In order to quantify the level and extent of infection in BALB/c and C57BL/6 mice, we were able to detect POWV RNA by qPCR in the brain, but not in the spleen, liver, kidney or cervical lymph node LN. The average POWV RNA genome copy number in the BALB/c brains was 1.5 x 10^6^ copies/μg of total RNA. The POWV RNA genome copy numbers in the brains of C57BL/6 mice were more varied, depending on severity and outcome of disease. The highest POWV RNA genome copy number was 3.3 x 10^7^, which was detected in the animal which was euthanized at 8 dpi (Fig 3D). The 2 animals that survived to the experimental end point of 28 dpi had lower viral genome copy numbers of 440 and 2175 copies/μg of total brain RNA, respectively (Fig 3D).

In marked contrast, i.p. inoculation of 4-week old *P*. *leucopus* mice with 10^3^ PFU of POWV resulted in no overt signs of clinical disease and all inoculated mice survived to the experimental end point of 28 days (Fig 3A). Furthermore, no evidence of illness was evident over the 28 day period of observation after i.p. inoculation of 10^2^, 10^5^ or 10^8^ PFU of POWV. In addition, no POWV RNA was detected by qPCR in the brain, spleen, liver or cervical LN samples harvested from i.p-inoculated *P*. *leucopus* mice at 28 days post challenge. Thus, these results suggested that POWV was not neuroinvasive in *P*. *leucopus* mice.

### Histopathology of *P*. *leucopus* mice inoculated with POWV

**Fig 4:**
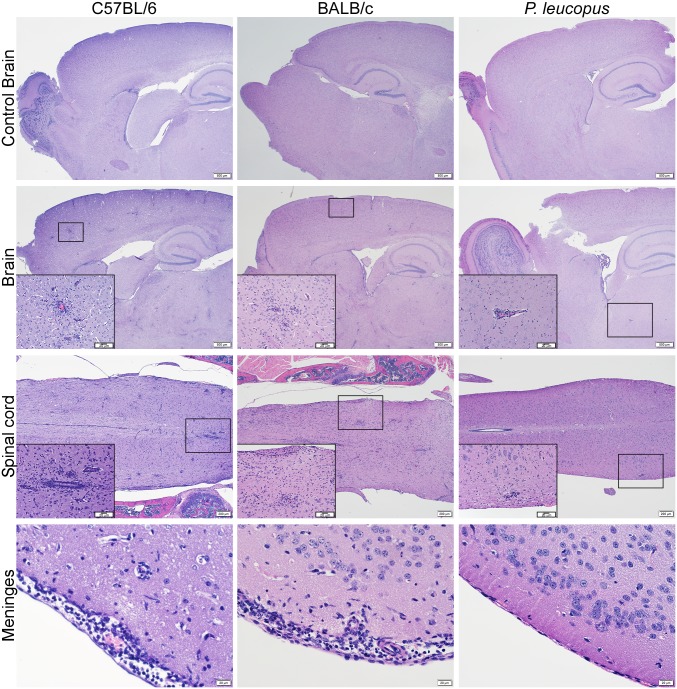
Hematoxylin and eosin-stained sections of brain and spinal cords obtained from i.c.-inoculated mice. The terminal brain sections of C57BL/6 (5 dpi) and BALB/c (4 dpi) mice show moderate lymphocytic perivascular cuffing and microgliosis, but these lesions were mild in *P*. *leucopus* brains (image obtained from mouse sacrificed at 15 dpi). *Peromyscus leucopus* spinal cords showed minimal lymphocytic perivascular cuffing and microgliosis, but BALB/c and C57BL/6 spinal cords showed moderate lesions. Normal meninges were observed for *Peromyscus leucopus*, but the meninges of BALB/c and C57BL/6 mice had moderate lymphocytic infiltrates.

To probe for subtle *P*. *leucopus* mouse responses to POWV, we examined sections of brain and spinal cord for any histological changes at various time points after i.c. inoculation with POWV. The histopathology revealed changes consistent with mild inflammatory responses in the brains and spinal cords of i.c.-inoculated *P*. *leucopus* mice from 5 to 15 dpi. The specific lesions were nodular microgliosis and lymphocytic perivascular cuffing (Fig 4), and these were uniform and more extensive at 15 dpi. However, no lesions were present at 28 dpi, suggesting that inflammation had resolved.

Examination of brain and spinal cord sections obtained from i.c.-inoculated BALB/c and C57BL/6 mice revealed the presence of lesions consistent with encephalitis and meningitis. In addition to nodular microgliosis and lymphocytic perivascular cuffing observed in *P*. *leucopus* mice, BALB/c and C57BL/6 mice also presented lymphocytic infiltrations in the meninges (Fig 4).

Taken together, these results suggested that POWV induced low-grade encephalitis in i.c.-inoculated *P*. *leucopus* mice. However, the inflammatory response was not associated with meningitis and seemed unaccompanied by observable clinical signs of illness. In contrast, BALB/c and C57BL/6 mice suffered severe, usually fatal, encephalitis, coupled with meningitis.

### Analysis of viral RNA distribution in the CNS by *in situ* hybridization

**Fig 5:**
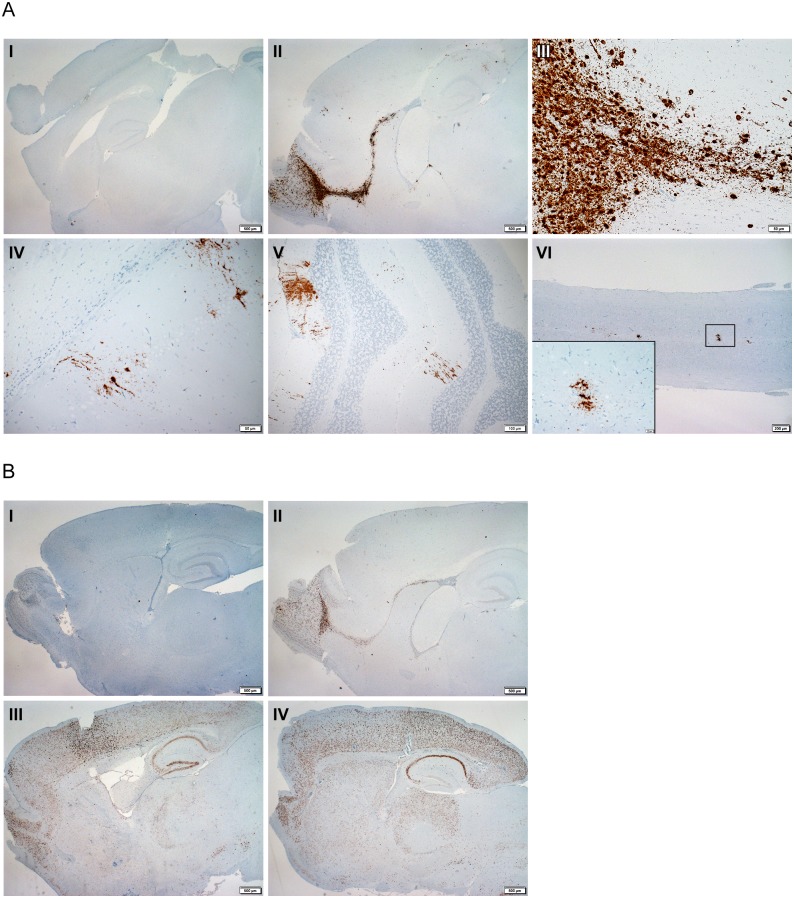
Distribution of POWV RNA in the CNS of i.c. inoculated determined by *in situ* hybridization. (**A**) POWV (+)RNA (stained brown) in the brain and spinal cord of i.c. inoculate *P*. *leucopus* mice. (**i**) Negative control showing no POWV (+)RNA. **(ii)** Diffuse detection of (+)RNA in the olfactory bulb (magnification x20). **(iii)** Close up image of the olfactory bulb (magnification x200). **(iv)** Multifocal detection of POWV (+)RNA in the paraventricular region of the brain. (**v**) Multifocal detection of POWV (+) RNA in the cerebellum. (**vi**) Multifocal detection of POWV (+)RNA in the spinal cord. (**B**) Detection of POWV negative RNA strand ((-)RNA) in the brains of *Peromyscus leucopus*, BALB/c and C57BL/6 mouse brains after ic inoculation. We used probes targeting the (-)RNA of POWV, which is only present during virus replication. (**i**) No viral RNA was detected in the mock-inoculated *P*. *leucopus* control. (**ii**) POWV (-)RNA was strongly and diffusely detected in the olfactory lobe of i.c. inoculated *P*. *leucopus*. (iii) Detection of diffuse POWV (-)RNA in the brain of an i.c. inoculated C57BL/6 mouse. (iv) Detection of diffuse POWV (-)RNA in the brain of an i.c. inoculated BALB/c mouse.

**Fig 6:**
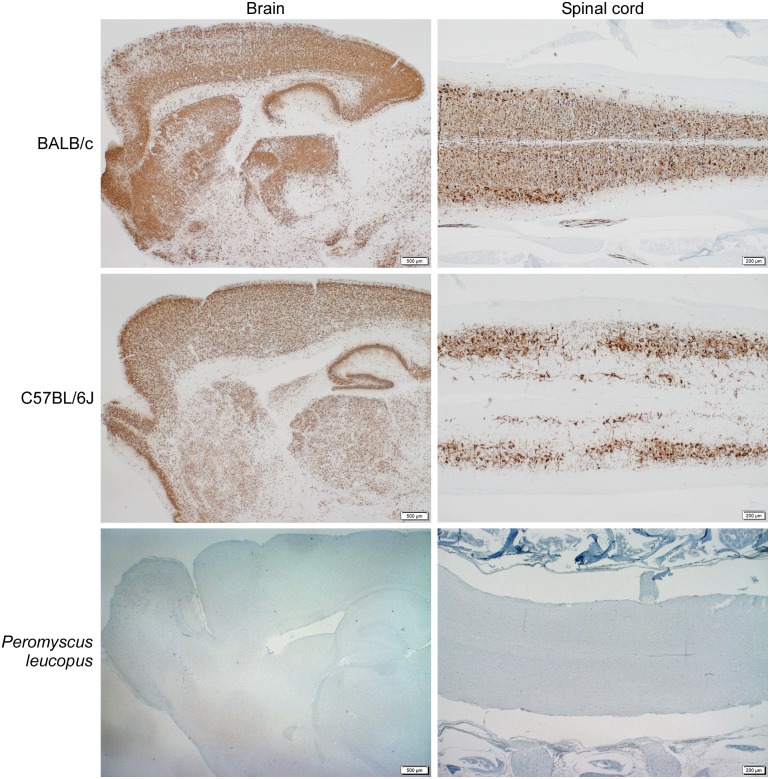
Detection of POWV (+)RNA in the brain and spinal cord by *in situ* hybridization after i.c. inoculation. Diffuse positivity was observed in the brains of both BALB/c and C57BL/6 mice at 4 or 5 dpi. Positivity in the spinal cord was diffuse in BALB/c mice, but multi-focal in C57BL/6 mice. No viral RNA could be visualized in the brain and spinal cord of *P*. *leucopus* mice at 28 dpi.

We used *in situ* hybridization (ISH) to analyze the distribution of POWV plus-sense genomic RNA in the CNS of *P*. *leucopus* mice over time, after i.c. inoculation, and small amounts of viral RNA were detectable at 1 dpi only in the subventricular white matter. From 2 dpi through to 7 dpi, POWV RNA was more abundant, but localized mainly to the olfactory bulb and ventricle (Fig 5A). Lesser amounts could be detected in the cerebral cortex, granular layer of the cerebellum and spinal cord (Fig 5A). However, the viral RNA was undetectable in *P*. *leucopus* CNS by ISH at 28 dpi (Fig 6). Calculations based on the qPCR results enabled us to estimate that the average POWV RNA copy numbers in the *P*. *leucopus* brains at 28 dpi (Fig 1) translated to ~0.004 genome copies per cell, suggesting that the viral load was too low for detection with ISH.

Next, we performed ISH analyses with a probe targeting the minus-sense strand of POWV RNA to determine if POWV was actually replicating in the brain of i.c-inoculated *P*. *leucopus* mice. POWV (-)RNA was detected and also localized mainly to the olfactory bulb and the ventricle (Fig 5Bii). Thus we confirmed that *P*. *leucopus* mice supported limited POWV replication.

In contrast, POWV plus-sense genomic RNA was widely distributed in most parts of the brain and the spinal cord in BALB/c and C57BL/6 mice (Fig 6). Minus-sense RNA was also distributed extensively in the brain (Fig 5Biii & 5Biv), indicating widespread permissive viral replication in these 2 mouse species.

### Serological response of *P*. *leucopus* to POWV challenge

To determine if *P*. *leucopus* mice challenged with POWV mounted an antibody response, we screened sera from i.c. and i.p. inoculated mice with an ELISA assay. The analysis showed that i.c.-inoculated *P*. *leucopus* mice generated anti-POWV antibodies in the titer range 250–1250. Further analysis indicated that these antibodies had a FRNT_60_ titer of 250. Thus, *P*. *leucopus* mice generated neutralizing antibodies when inoculated i.c. However, anti-POWV antibodies were undetectable in mice that had been i.p. inoculated, except those that got 10^8^ PFU of POWV (titer 250).

## Discussion

Powassan virus (POWV) causes severe encephalitis and death in humans in North America and some parts of Russia [1, 5, 6, 8, 9, 44, 45]. The virus persists in a cycle involving infected ixodid tick vectors, and small to medium-sized mammals, which play important roles as reservoirs, amplification or bridge hosts [1, 26–28]. In this study, we have examined POWV infection in 2 strains of laboratory mice (BALB/c and C57BL/6), and a strain of wild mice (*P*. *leucopus*), which might be a natural host for this virus.

POWV was neurovirulent and neuroinvasive in both strains of laboratory mice, causing a severe neurological disease that was uniformly fatal in BALB/c mice, but killed only 60% of C57BL/6 mice. Thus, these 2 strains of mice were suitable models for the human disease [36, 37, 46]. In marked contrast, the *Peromyscus* showed no evidence of virus replication or disease after peripheral inoculation, although mice inoculated with 10^8^ PFU developed a modest antiviral antibody response. Notably, although i.c. inoculation of POWV into the *Peromyscus* mice did not result in observable disease, there was clear evidence of low level viremia and limited viral replication in the olfactory bulb that was cleared by 28 dpi. Thus, the biology of this virus in the natural host is very different from that in laboratory mice, and our findings may have implications for the role of the natural host in the ecology of POWV and other pathogens that are found in these mice [38, 40].

Low-level viremia was observed only at early time points of experimental infection in *P*. *leucopus* mice. This may be the critical time period during which the ticks could acquire virus from feeding on a host under these experimental conditions. Natural infection of mammals occurs via tick-bites, and the tick saliva has been shown to enhance transmission of POWV and affects the course disease [37]. However, co-feeding is probably the major mechanism by which TBFVs are transmitted between ticks during feeding [20, 21, 23, 24].

Intraperitoneal inoculation of 4-week old BALB/c mice was uniformly lethal in our laboratory. Interestingly, C57BL/6 mice were partially resistant to i.p challenge with POWV and the surviving mice still had viral RNA in the brain tissue, suggesting viral persistence. This result was similar to the effect of the mosquito-borne West Nile virus (WNV) in C57BL/6 mice inoculated subcutaneously in which the virus caused 20% mortality [47]. We and others previously showed that C57BL/6 mice are partially resistant to the attenuated TBFV Langat virus when inoculated i.p. [48, 49], suggesting that a similar mechanism of resistance may be involved. However, one report recently described 100% mortality in C57BL/6 mice inoculated with 5 x 10^3^ or 5 x 10^6^ PFU of POWV via footpad injection [46]. It is unclear if these differences result from the route of infection, source of mice, differences in the POWV, or some other undefined variable. Nevertheless, C57BL/6 may be useful as a model to study flavivirus persistence [47].

*P*. *leucopus* mice were completely refractory to POWV infection following i.p. inoculation although mice injected with extremely high doses developed POWV specific antibodies. And even then, there was no sign of disease or viral replication in the organs that we surveyed. We note that our experimental infection differs completely from natural infection through tick bites and this could be the reason why we do not observe seroconversion in i.p. inoculated *P*. *leucopus* mice. The differences include the fact that the ixodid ticks feed slowly for 3–5 days while inoculating immunomodulating salivary components, and each mouse may be infested with several ticks at the same time. As a result, the continued exposure to virus in the presence of tick salivary components over time is likely to lead to a robust seroconversion in nature. Thus, we hypothesize that the *P*. *leucopus* mice may be quickly and efficiently restricting i.p. inoculated POWV before a humoral immune response. In pursuit of this hypothesis, we are currently studying potential restriction factors that could be involved in restriction of POWV.

We also did not observe obvious signs of disease following i.c. inoculation of *P*. *leucopus* mice with POWV, but mild encephalitis developed as indicated by the presence of minimal to moderate lymphocytic perivascular cuffing and nodular microgliosis in the CNS. The encephalitis had resolved by 28 dpi although viral RNA was still detectable at this time point. Our results were similar to observations by Telford et al [40], who reported that adult *P*. *leucopus* mice challenged subcutaneously with the DTV, a close relative of POWV, survived with no apparent illness. Similar observations were also made in the bank vole (*Myodes glareolus*), a natural host of tick-borne TBEV in Europe [50], but some bank voles developed acute generalized symptoms at 8 dpi [50], an observation not seen in *P*. *leucopus* mice. In contrast, 67% (2/3) of bank voles infected with TBEV exhibited inflammatory changes in the brain at 25 dpi [50], suggesting that resolution of encephalitis caused by TBEV may take longer in the bank vole. Taken together, the emerging picture is that natural rodent hosts do not suffer the uniformly severe disease characteristic of human infection or that in laboratory mice.

The accumulation of POWV (+)RNA and (-)RNA was limited to the olfactory bulb and ventricle of i.c. inoculated *P*. *leucopus* mice brains with minimal amounts in other regions (Fig 5). In Swiss albino, C3H and C57BL/6 mice, the initial detection of viral antigen reportedly is in the olfactory bulb following intranasal, subcutaneous or i.p. inoculation of neurotropic flaviviruses [51, 52]. It has been proposed that the olfactory bulb may be more permissive for viral replication in these mouse species, and that viremia may contribute towards increased virus replication across the whole brain [51, 53]. Our results showed that POWV rapidly replicated and spread across the brains of i.c. inoculated BALB/c and C57BL/6 mice by 4 dpi. However, this was never the case for *P*. *leucopus* mice (Figs 5 & 6) even though viremia was evident in the first 4 dpi. Thus, when compared to BALB/c and C57BL/6 mice, *P*. *leucopus* mice are clearly capable of limiting virus replication and spread in the brain.

The limited extent of infection in *P*. *leucopus* mice suggests that these mice are able to restrict replication and spread of POWV. Some restriction factors operative against various vector-borne flaviviruses have been described [54–56]. A report by Kurhade et al [49] suggests that IPS-1 signaling is important for controlling LGTV replication in the brains of C57BL/6 mice [49], and this has also been shown to be true for Japanese encephalitis virus and WNV replication in BALB/c mouse brains [57, 58]. The *Peromyscus* genus is divergent from the *Mus* genus [59], and it remains to be determined if IPS-1 signaling and restrictions factors, such as TRIM79α [54] are important in the *P*. *leucopus* mouse. Therefore, additional research aimed at understanding the molecular mechanisms of peripheral and CNS restriction of POWV in *P*. *leucopus* is warranted. Experiments designed to model POWV restriction in cell cultures systems may prove informative.

In conclusion, *P*. *leucopus* mice do not show clinical signs of disease after i.c. or i.p. inoculation with POWV. However, POWV induces minimal to mild encephalitis without meningitis at early time-points and inflammation resolves by 28 dpi following i.c. inoculation. *P*. *leucopus* mice restrict POWV replication mainly to the olfactory bulb and ventricle without extensive spread to the whole brain, suggesting that these mice have restriction factors, which need to be further characterized. The *P*. *leucopus* mouse is a novel model, which will be useful for studying efficient host responses and molecular mechanisms of effective restriction of POWV and other viruses in the TBEV serogroup.
